# Screening for Beta Thalassemia Carrier State Among Women Attending Antenatal Clinic in a Tertiary Care Centre and Framing a Model Program for the Prevention of Beta Thalassemia

**DOI:** 10.7759/cureus.22209

**Published:** 2022-02-14

**Authors:** Febe Renjitha Suman, Ravi Teja, Jesu Magdalene, Tejaswita Bisht, Suresh Varadharajan, Uma Lakshmi, Jayalakshmi Balasubramanian

**Affiliations:** 1 Pathology and Laboratory Medicine, Sri Ramachandra Institute of Higher Education and Research, Chennai, IND; 2 Pathology, Sri Ramachandra Institute of Higher Education and Research, Chennai, IND

**Keywords:** thalassemia, mentzer index, microcytic hypochromic anemia, high performance liquid chromatography, hemoglobinopathy

## Abstract

Aim

This study was carried out to detect beta (β) thalassemia heterozygous state in antenatal women and to create a validated flag in the software utilizing the screening indices to filter the samples to be subjected to high-performance liquid chromatography (HPLC) and to define a model for the prevention of thalassemia.

Methods

This cross-sectional study was carried out for a period of two years on women attending the antenatal clinic. Complete blood count (CBC) and peripheral smear were done during their first visit. Serum iron and total iron-binding capacity were done for women who had microcytic hypochromic anemia. The samples of women without iron deficiency were processed by HPLC for hemoglobinopathies. The spouses of women who were found to have beta thalassemia trait were counseled to undergo screening, and those who consented were tested.

Results

A total of 183 antenatal women were screened for hemoglobinopathies. Βeta thalassemia trait was detected in 23.5% of them. Among the 16 red blood cell (RBC) indices analyzed, Sehgal index and Mentzer index, both with sensitivities of 97.67%, were found to be suitable. Alert flag incorporated in the software of the analyzer to detect these indices helps not to miss samples to carry out HPLC. The spouses of women with β thalassemia trait who underwent HPLC testing were 55.81%. A model screening program was designed.

Conclusion* *

Antenatal testing by HPLC should be done on all mothers having microcytic hypochromic anemia without iron deficiency. Spouse testing of the woman who was carriers denotes the success of the antenatal screening program.

## Introduction

Anemia is a common disease found in females, especially during pregnancy, with a prevalence of about 54% [1]. Anemia is due to globin deficiency is refractory to iron supplements. The globin deficiencies include hemoglobinopathies caused by genetic mutations affecting the genes, commonly thalassemias. 

Thalassemias are autosomal recessive disorders consisting of homozygous and heterozygous variants. While the homozygous or compound heterozygotes state results in clinically significant phenotypes of variable severity, heterozygous are symptom-free but present with hematological characteristics often useful for their identification. Due to the high prevalence of consanguineous marriages in underdeveloped and developing countries, the homozygous state of hemoglobinopathies tends to be higher [2]. As the morbidity, mortality, and economic burden are high in beta thalassemia; it is essential to identify carriers in order to assess the risk of a couple giving birth to a severely affected child with thalassemia. 

There are several modalities available to prevent the birth of a thalassemia-affected child. Due to the existing social and cultural taboos, the scope for detecting thalassemia before conception or marriage is limited though it is the ideal method. Hence screening during pregnancy is preferred [3]. 

Screening for thalassemia is carried out to detect the carrier state among the couples. The laboratory methods for carrier identification include red blood cell (RBC) parameters and peripheral smear examination. The popular and most widely used Mentzer index can be used as a relatively inexpensive calculation; however, confirmatory tests are done to detect hemoglobin variants [4]. Though there are various methods like high-performance liquid chromatography (HPLC), globin chain analysis, and DNA analysis, the simple and economic method of HPLC is ideal for detecting most of the beta chain variants. 

The purpose of the antenatal screening program is to identify the carrier states and also the probability of high-risk pregnancy. The success of an effective screening program is to provide counseling and enable the couples to make informed decisions. A primary screening approach with carrier identification strategies may provide guidance on whether to include thalassemia screening as a mandatory investigation in the initial laboratory tests done for antenatal women in the particular population.

## Materials and methods

This study was carried out as a cross-sectional study in the department of pathology of our tertiary care hospital for a period of two years (2018-2020). The institutional ethics committee approved the proposal, and informed consent was obtained from all study participants (IRB-CSP-MED/15/AUG/24/09). The study group consisted of antenatal women from six to 12 weeks gestational age, attending our tertiary care facility and having microcytic hypochromic anemia. Women who had iron deficiency, as well as women who were not willing for counseling and HPLC testing, were excluded. 

Complete blood count was analyzed using Beckman Coulter LH 780 (Beckman Coulter, Inc., Brea, CA, USA). Hemoglobin (Hb), red blood cell count (RBC), mean corpuscular volume (MCV), mean corpuscular hemoglobin (MCH), mean corpuscular hemoglobin concentration (MCHC), hematocrit and red cell distribution were observed. Peripheral smear was also examined to look for microcytic hypochromic RBCs and target cells.

The blood samples of women with microcytic hypochromic anemia (MCV<80 fL, MCH< 27pg, Hb<11g/dL) were subjected to iron studies. Those samples which were not deficient in iron were processed for hemoglobin variants by HPLC using D10 Haemoglobin Testing System (Bio-Rad Laboratories, Inc., Hercules, CA, USA). Hb A2 of 3.5% and below were considered normal, and 3.6% and above were considered beta thalassemia trait. Potential sources of variation were also considered [5,6].

Standard descriptive analysis was done on demography and ethnicity. The sensitivity and specificity of all the indices to predict β thalassemia trait were analyzed using R base software version 3.4.2 for Mac. The sensitivity, specificity, and receiver operating characteristic (ROC) curves were computed using the pROC package for R software. The higher sensitivity and specificity were taken from the ROC curve as a cutoff value for each index. 

## Results

This prospective study included samples from 183 antenatal women that were processed for hemoglobin variant analysis by HPLC. 

The age of these antenatal women ranged from 18 - 40 years, and they were between six to 12 weeks of pregnancy.

Out of the 183 samples processed by HPLC, 43 (23.5%) women were diagnosed with β thalassemia trait. Samples from 24 (13.1%) antenatal women were diagnosed as having other hemoglobin variants. One hundred and sixteen (63.4%) samples were normal. Equivocal value had been derived in none. 

Among women with β thalassemia trait, 25.6% belonged to the age group of 18-23 years, 39.5% were in the group of 24-29 years, 27.9% were in the group of 30-35 years, and 7.0% were in the group of 36-40 years.

The hemoglobin ranged from 7.8 to 10.8 g/dl (mean=8.9 g/dl). The RBC counts were between 3.82 million/μl and 5.89 million/μl (mean=5.21 million/μl). MCV ranged from 50.3 to 70.9 fl (mean=65.12 fl) and MCH was 13.2 to 27.1 pg (mean=20.86 pg). 

**Figure 1:**
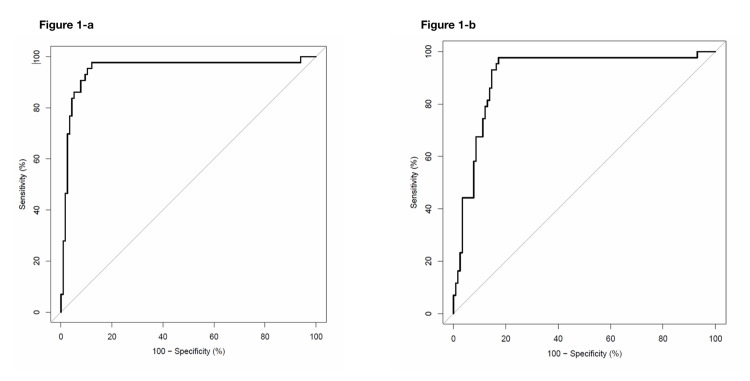
ROCs for Mentzer Index (A) and Sehgal Index (B)

Various RBC indices and formulas to discriminate β thalassemia trait from iron deficiency anemia were calculated, and the sensitivity and specificity of each of the values were derived. A threshold at which the highest sensitivity and specificity for the data in the current study sample was obtained from the statistical analysis and ROC curves for each of the indices. The data is shown in Table 1, and the ROC curves for Mentzer and Sehgal index are shown in Figure 1.

**Table 1:** Comparison of threshold, sensitivity and specificity of the original studies with the present study

Index	Original threshold	Sensitivity	Specificity	Present threshold	Sensitivity	Specificity
Menzter [4]	<13	76.74	95.68	<14.1	97.67	87.93
Red cell volume distribution width index (RDWI) [7]	<220	79.069	96.55	<255.9	93.023	93.10
Shine & Lal [8]	<1530	97.67	47.41	<1142.41	95.34	66.37
Srivastava [9]	<3.8	37.20930	96.55	<4.69	95.34	79.3
Green & King [10]	<65	46.51	97.41	<84.9	97.6	90.5
Sirdah [11]	<27	39.53	99.13	<33.74	97.67	88.79
Ehasani [12]	<15	74.41	93.1	<20.1	97.67	85.34
England & Fraser [13]	<0	16.27	99.13	<10.98	97.6	91.3
Ricera [14]	<4.4	93.02	67.241	<3.65	88.37	87.06
Mean density of Hb/liter of blood (MDHL) [15]	>1.63	60.46	98.27	>1.48	88.37	96.55
Mean cell Hb density (MCHD) [15]	>0.3045	76.74	27.58	>0.3436	18.60	98.2
Sehgal [16]	<972	97.67	82.75	<971.55	97.67	82.75
Red blood cell (RBC) Count	>4.81	86.04	95.6	>4.78	88.37	95.68
Mean corpuscular volume (MCV)	<80	97.67	39.65	<70.45	97.67	68.1
Mean corpuscular hemoglobin (MCH)	<27	36.2	95.34	<23.7	93.02	56.03
Red cell volume distribution width (RDW)	<18	86.04	48.27	<17.35	83.72	53.44

Testing for hemoglobin variants by HPLC was done for 24 (55.81%) of the spouses of the women who are carriers for beta thalassemia.

## Discussion

Hemoglobinopathies are the most common hereditary disorder in the world. It has been estimated that about 7% of the world's population carries an abnormal hemoglobin gene, and about 3% carry a β thalassemia gene [17,18]. The frequencies of carriers in the Indian population ranges from 8.9% to 37.9% [19,20]. Very few studies are available in Tamil Nadu, and Coimbatore, Ooty, and Dharampuri are considered hot spots. Tamil Nadu is said to be known for the consanguineous marriages, and children with thalassemia born to first cousins were far higher than others [21]. A study done in 2014 estimated about 7.04% beta thalassemia trait among the population of Chennai [22]. Chennai being a metropolitan society, has vast number of migrant population from across the state and the country. Inter-caste and inter-religious marriages are common despite the prevalence of consanguinity. Hence the study place is selected. People of high to low income groups are included as the medical college hospital caters to the low-income group.

In this study, 24% of our patients were carriers of beta thalassemia. All the patients were anemic of microcytic hypochromic type. Table 1 compares the sensitivity and specificity values of the RBC discriminatory indices for β thalassemia trait in this study with sensitivity and specificity values from the original studies. 

Mentzer index (MI), an index used worldwide to discriminate β thalassemia trait, had a sensitivity of 76.44% and a specificity of 95.68% when the cut-off was <13. But the sensitivity was higher (97.67%) with a cut-off of <14.1. The lower sensitivity of 60% and higher specificity of 93.1% were detected with MI <13 in pregnant women of Rajasthan [21]. However, the study done in Mumbai was in concordance with the present study at cut-offs of <13 and <14 [16]. Similar sensitivity was observed in Turkey and Iran at a cut-off of <13 [23,24]. High sensitivity was noted by studies from Punjab and Thailand [22,25]. A study done in Tamil Nadu had a sensitivity of 93.3% but a lower specificity at 52.9% [26]. The Mentzer index at a higher cut-off of 14< may be suitable for Indians.

Sehgal index was developed in India in 2015. This index utilized red cell volume and count. The original study observed a sensitivity of 89.74% and a specificity of 86.96% with a cut-off of <972 for the β thalassemia trait, with a high Youden index of 76.7 [16]. In the present study, at the same cut-off, higher sensitivity and specificity of 97.67% and 82.75%, respectively, were obtained. This index and Mentzer index at a higher cut-off may be promising screening RBC indices. However, multicentric studies may be done to authenticate the same.

Ehsani Index, Sirdah index, England & Fraser index, Green & King had very high sensitivity and reasonably high specificity [10-13]. Red cell volume distribution width index (RDWI), mean corpuscular volume (MCV), mean corpuscular hemoglobin (MCH), Shine & Lal, and Srivastava index are highly sensitive but not very specific. Ricerca index had reasonably good sensitivity and specificity. RBC count, the mean density of Hb/liter of blood (MDHL), mean cell Hb density (MCHD) are more specific but not very sensitive.

Among the 43 women found as carriers by HPLC, 30 (69.7%) were from Tamil Nadu. The other women are migrant population, including 10 (23.2%) from West Bengal, two from Andhra Pradesh, and a single person from Assam. These observations state that it is necessary to do antenatal screening to reduce its burden in Tamil Nadu, which was thought to have a lower prevalence of thalassemia.

Spouse testing, the next step in the prevention of thalassemia, was done for 24 men (55.8%). The reasons for others not to be tested included prior knowledge of their personal chromatogram, logistic reasons and/or hesitance. 

**Figure 2:**
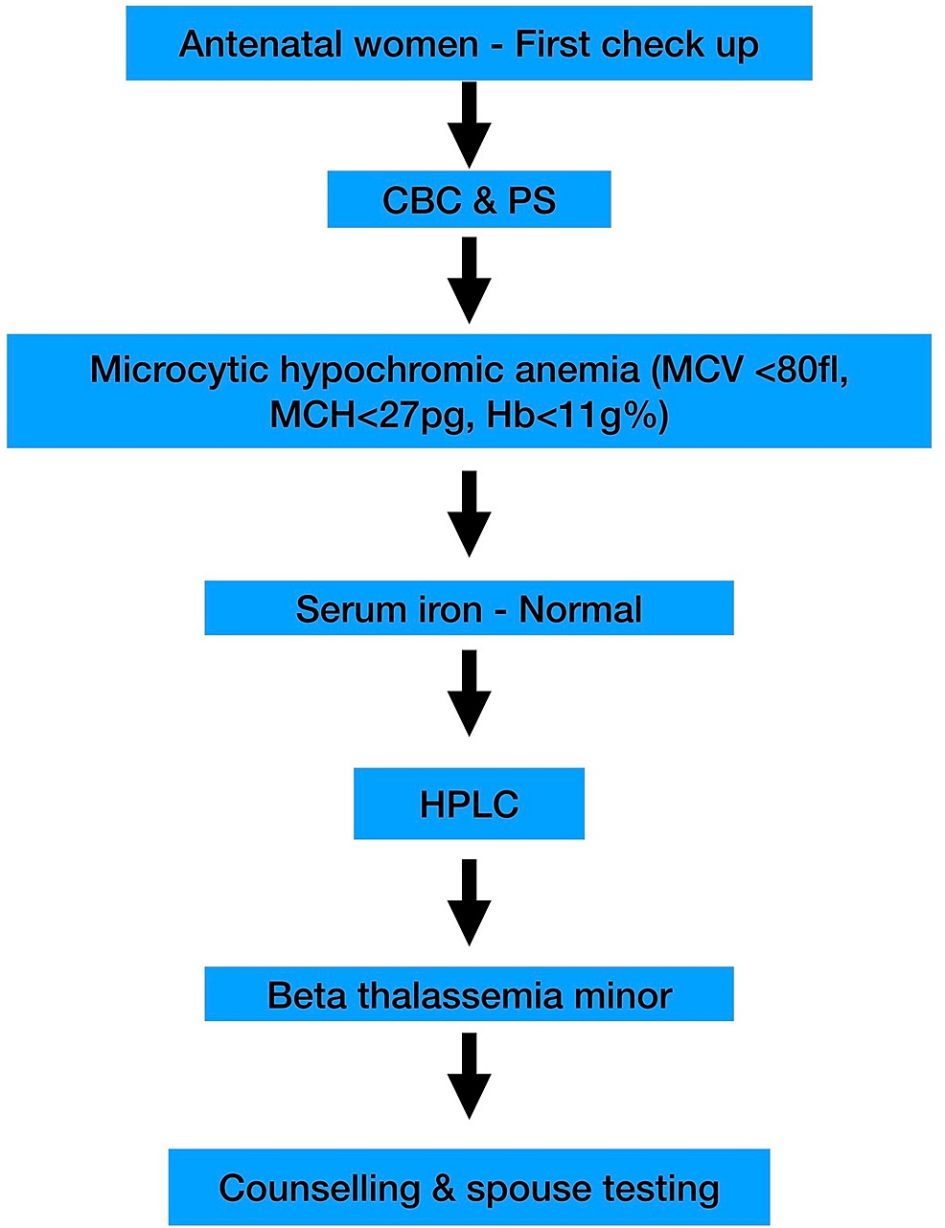
Model screening program CBC - complete blood count; PS - peripheral smear; MCV - mean corpuscular volume; MCH - mean corpuscular hemoglobin; HPLC - high-performance liquid chromatography

The sensitivity and specificity of all the RBC indices and mathematically calculated indices established by experts for screening of thalassemia were analyzed. Sehgal index at a cut-off <972, Mentzer index at a higher cut-off of <14 may together be promising RBC screening indices. In laboratories, where hematology analyzers are available, a validated flag rule of Mentzer index or Sehgal index is implemented as a pop-up window to alert suspected cases of β thalassemia trait. Though the β thalassemia trait can be suspected based on validated indices, HPLC is required for confirmation. Equivocal results need genetic testing. Model screening program designed based on this study, as shown in Figure 2, may help in the prevention of thalassemia.

## Conclusions

This approach will help in appropriate screening and detection of women who are carriers. Spouse testing and counseling of couples at risk will reduce the morbidity and mortality from a potential homozygous offspring. This investigation can be implemented in private and government tertiary care centers, and they can cater samples from primary and secondary health centers. 
